# Efficacy and safety of local consolidative therapy combined with systemic therapy in driver-negative oligometastatic non-small cell lung cancer: a systematic review and meta-analysis

**DOI:** 10.3389/fonc.2026.1820515

**Published:** 2026-04-20

**Authors:** Heqiao Han, Lian Li, Taiwon Park, Yuzhao Yang, Guha Alai, Chuan Zhong, Yidan Lin

**Affiliations:** 1 Thoracic Surgery department, West China Hospital, Sichuan University, Chengdu, China; 2 Outpatient department, West China Hospital, Sichuan University, Chengdu, China

**Keywords:** driver-negative, immunotherapy, local consolidative therapy, meta-analysis, oligometastatic non-small cell lung cancer

## Abstract

Oligometastatic non-small cell lung cancer (NSCLC) represents a potentially modifiable stage of metastatic disease, however, the survival benefit of local consolidative therapy (LCT) in patients without actionable driver mutations remains uncertain in the era of immunotherapy. To address this clinically relevant controversy, we performed a systematic review and meta-analysis evaluating the efficacy and safety of adding LCT to systemic therapy in driver-negative oligometastatic NSCLC. A comprehensive search of PubMed, Embase, and the Cochrane Library through December 2025 identified 13 eligible studies including 1,698 patients. Pooled analyses demonstrated that the addition of LCT was significantly associated with improved overall survival (HR 0.45, 95% CI 0.36-0.56) and progression-free survival (HR 0.53, 95% CI 0.46-0.61) compared with systemic therapy alone. Notably, cumulative meta-analysis showed that the survival advantage remained stable as evidence accrued over time, underscoring the consistency of the treatment effect. LCT was also associated with a higher objective response rate (RR 1.47, 95% CI 1.12-1.93) without a significant increase in treatment-related adverse events (RR 1.31, 95% CI 0.93-1.84). Importantly, subgroup analyses demonstrated that the survival benefit persisted in studies incorporating immunotherapy-based regimens, suggesting that LCT retains clinical value even within contemporary systemic treatment paradigms. Sensitivity analyses confirmed the robustness of these findings, and no significant publication bias was detected. Collectively, these results indicate that integrating LCT into systemic therapy is associated with meaningful survival gains in driver-negative oligometastatic NSCLC without added toxicity, supporting a risk-adapted, multidisciplinary treatment strategy in the modern immunotherapy era.

**Systematic Review Registration:** https://www.crd.york.ac.uk/prospero/, identifier CRD420261351878.

## 1 Introduction

Non-small cell lung cancer (NSCLC) remains the leading cause of cancer-related mortality worldwide (1). Among patients with advanced disease, those presenting with oligometastatic involvement, typically defined as a limited number of metastatic lesions, are increasingly recognized as a biologically and clinically distinct subgroup (2, 3). Traditionally, systemic therapy has been regarded as the cornerstone of treatment for this population. However, accumulating evidence suggests that the addition of local consolidative therapy (LCT), including surgery, radiotherapy, or other ablative approaches, may further improve disease control and survival outcomes (4–7). On this basis, a growing number of prospective and retrospective studies have explored and supported the potential role of LCT in oligometastatic NSCLC. Nevertheless, the optimal integration of LCT into contemporary treatment paradigms remains a matter of ongoing debate, and current clinical guidelines offer inconsistent or cautious recommendations, resulting in substantial uncertainty in routine clinical decision-making (8–10).

Importantly, the perceived benefit of LCT has recently been questioned by emerging randomized evidence in the immunotherapy era. The NRG-LU002 phase II/III trial reported no significant improvement in progression-free or overall survival with the addition of local radical treatment to maintenance systemic therapy among patients with oligometastatic NSCLC who achieved at least stable disease after induction treatment, a population in which approximately 90% of patients received immunotherapy-based regimens (11). These results have prompted renewed scrutiny of the incremental value of local treatment in the setting of contemporary systemic therapy and have intensified ongoing debates regarding the optimal management of synchronous oligometastatic disease, particularly in patients without oncogene-addicted tumors.

Despite extensive investigation, clinically actionable conclusions remain elusive, largely owing to several structural limitations in the existing literature (12). First, much of the available evidence has been derived from molecularly selected populations, particularly patients harboring actionable driver mutations who derive substantial benefit from targeted therapies (13–18). In contrast, patients with driver-negative NSCLC, whose systemic treatment predominantly relies on chemotherapy and/or immunotherapy, have been relatively underrepresented. This imbalance limits the generalizability of prior findings to this large and clinically relevant subgroup.

Second, previous studies and meta-analyses have frequently focused on a single modality of local consolidative therapy, most commonly stereotactic body radiotherapy, while overlooking the broad spectrum of local treatment strategies routinely employed in real-world clinical practice (19, 20). This narrow focus, together with heterogeneous study designs, variable patient selection, and inconsistent outcome definitions, has substantially constrained the interpretability and clinical applicability of the existing evidence.

Therefore, despite a growing body of research, the true survival benefit and safety profile of LCT in patients with driver-negative oligometastatic NSCLC remain uncertain. A comprehensive synthesis of available evidence that directly addresses these key limitations is urgently needed. In the present study, we performed a systematic review and meta-analysis to evaluate the efficacy and safety of adding LCT to systemic therapy in patients with advanced oligometastatic NSCLC without actionable driver mutations. By integrating all available forms of LCT and focusing on a population in which targeted therapy does not dominate treatment decisions, this analysis seeks to provide robust and clinically relevant evidence to inform patient selection and optimize multidisciplinary treatment strategies.

## 2 Methods

This systematic review and meta-analysis has been registered in the PROSPERO database (registration number: CRD420261351878).

### 2.1 Search strategy and study selection

A systematic literature search was conducted in PubMed, Embase, and the Cochrane Library from database inception to December 2025, in accordance with the Preferred Reporting Items for Systematic Reviews and Meta-Analyses (PRISMA) guidelines. The search strategy combined terms related to non-small cell lung cancer, oligometastatic disease, and local consolidative therapy, including surgery, radiotherapy, and ablative techniques. Two investigators independently screened titles and abstracts for eligibility. Full texts of potentially relevant studies were subsequently reviewed. Discrepancies were resolved by consensus or consultation with a third reviewer.

### 2.2 Eligibility criteria

Studies were considered eligible if they met the following criteria: (1) included patients with advanced (stage IV) non-small cell lung cancer presenting with oligometastatic disease, as defined by each study. Oligometastatic disease was further categorized, when applicable, as synchronous (at initial diagnosis), oligo-residual (after systemic therapy), or metachronous (after prior curative-intent treatment), according to the timing of metastasis; (2) focused on patients without actionable driver mutations such as EGFR, ALK, ROS1, or provided extractable data specifically for driver-negative subgroups; (3) compared systemic therapy combined with LCT versus systemic therapy alone; and (4) reported at least one relevant outcome, including overall survival (OS), progression-free survival (PFS), objective response rate (ORR), or treatment-related adverse events (TRAE).

Studies were excluded if they: (1) exclusively included patients with actionable driver mutations; (2) did not allow separation of driver-negative data from mixed populations; (3) were reviews, case reports, conference abstracts without sufficient data, or non-human studies; or (4) lacked sufficient data for extraction.

### 2.3 Data extraction and outcome definitions

Data were independently extracted by two reviewers using a standardized data collection form. Extracted variables included: study characteristics (author, year, study design), patient demographics, systemic treatment regimens, type and extent of local therapy, follow-up duration, and reported outcomes. The primary outcome was OS and PFS. Secondary outcomes included ORR and TRAE. When HRs and corresponding 95% confidence intervals (CIs) were not directly reported, they were estimated from available survival curves using established methods.

### 2.4 Quality assessment

The methodological quality of RCTs was assessed using the Cochrane Risk of Bias tool, while observational studies were evaluated using the Newcastle–Ottawa Scale (NOS).

Studies with a NOS score ≥6 were considered to be of moderate to high quality. Quality assessment was performed independently by two reviewers, with disagreements resolved by consensus.

### 2.5 Statistical analysis

Hazard ratios (HRs) were used as summary measures for time-to-event outcomes, while risk ratios (RRs) were applied for binary outcomes. All effect estimates were pooled using the inverse variance method. Given the anticipated clinical and methodological heterogeneity across studies, such as the heterogeneity in LCT modalities, random-effects models were applied for all primary analyses. Statistical heterogeneity was assessed using the I² statistic, with values >50% indicating substantial heterogeneity. Meanwhile, cumulative meta-analyses were conducted to assess the temporal stability of the overall treatment effect of LCT as a therapeutic strategy, rather than to evaluate modality-specific effects. All statistical analyses were performed using R software (version 4.5.2) with the given packages. A two-sided p value <0.05 was considered statistically significant unless otherwise specified.

### 2.6 Subgroup and sensitivity analyses

Prespecified subgroup analyses were performed according to: (1) use of immunotherapy in systemic treatment (IO vs non-IO); and (2) extent of local therapy (all lesions treated, partial lesions treated, or mixed strategies).

Sensitivity analyses were conducted using leave-one-out methods to evaluate the robustness of pooled estimates.

### 2.7 Assessment of publication bias

Publication bias was evaluated by visual inspection of funnel plots and formally assessed using Egger’s regression test. A p value <0.10 was considered suggestive of small-study effects.

## 3 Results

### 3.1 Study selection and characteristics

**Figure 1:**
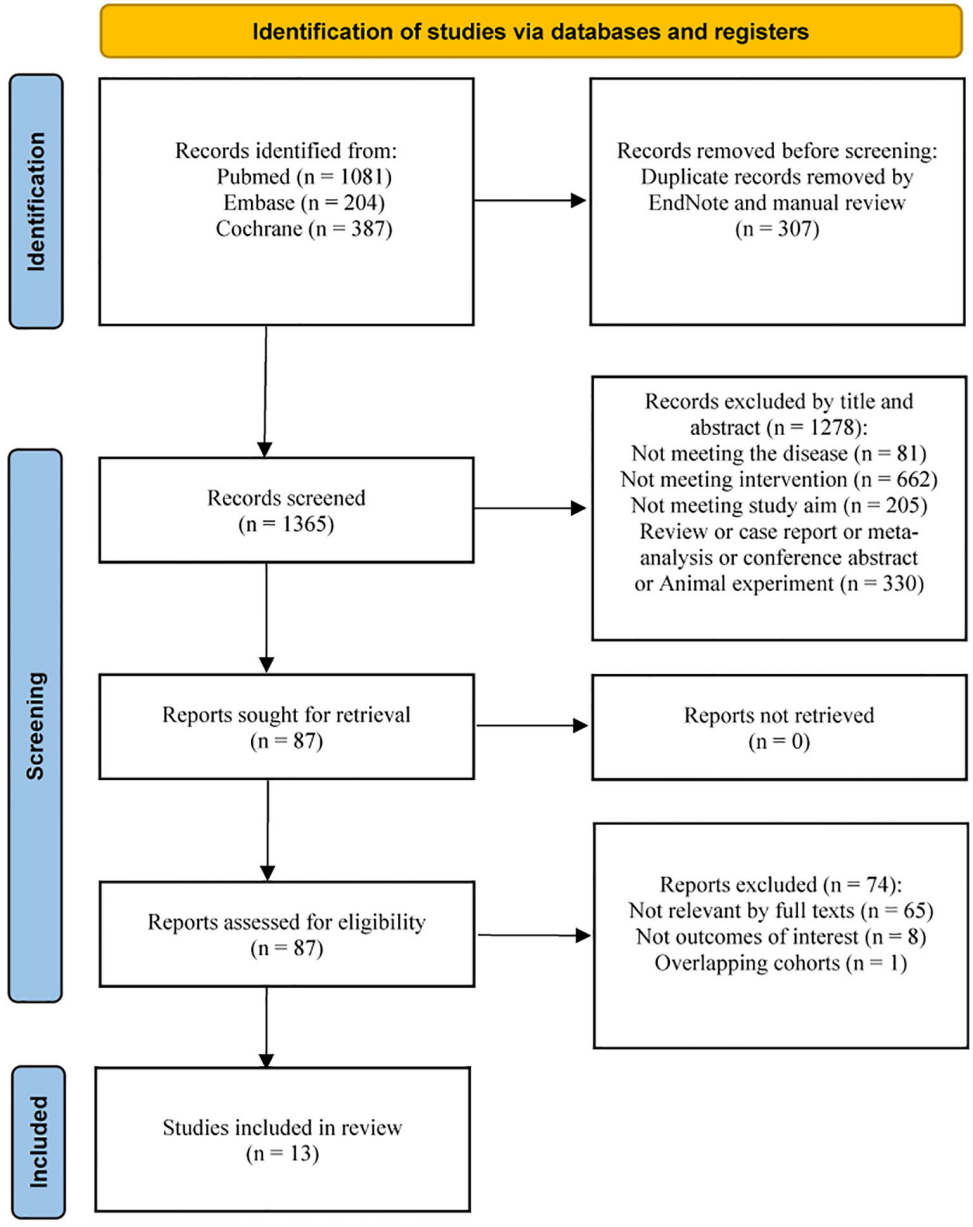
PRISMA flow diagram of study identification and selection. Flowchart illustrating the identification, screening, eligibility assessment, and inclusion of studies in this systematic review and meta-analysis. A total of 13 studies were ultimately included in the quantitative synthesis.

A total of 13 studies encompassing 1698 patients with advanced oligometastatic NSCLC without actionable driver mutations were included in the final meta-analysis (**Figure 1**) (6, 21–32). The RCT quality assessment plots and summary plots are presented in **Supplementary Figures 1**, **2**, respectively. The NOS scores of the retrospective cohort studies are presented in **Supplementary Table 1**, with the vast majority being high-quality studies. Across the included studies, patients received systemic therapy alone or systemic therapy combined with local consolidative therapy (LCT), including surgery, radiotherapy, or other approaches (**Table 1**). Overall survival (OS), progression-free survival (PFS) were the primary outcomes, while objective response rate (ORR), and treatment-related adverse events (TRAE) were the secondary outcomes of interest.

### 3.2 Overall survival

**Figure 2:**
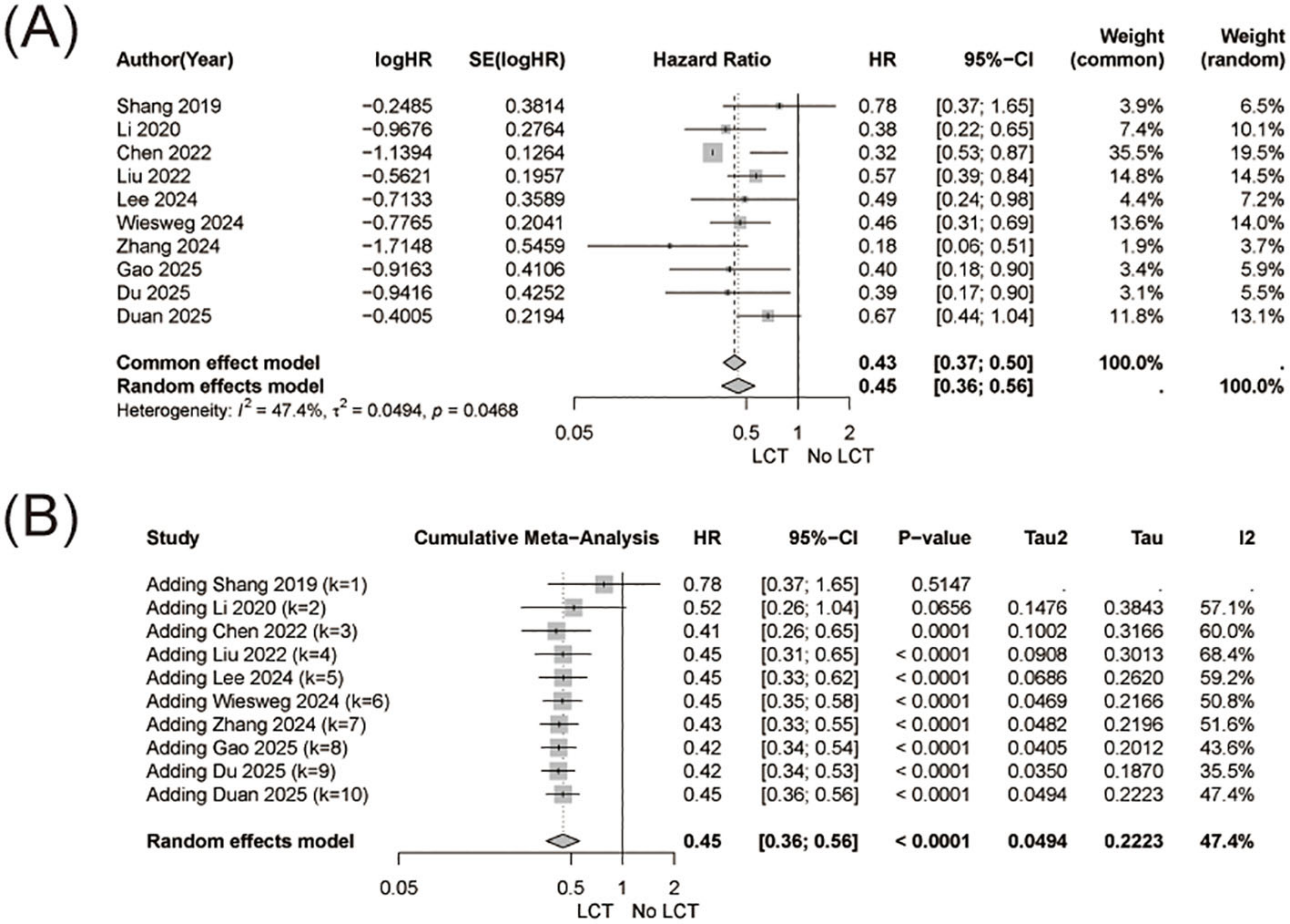
Overall survival (OS) analysis. **(A)** Forest plot showing pooled hazard ratios (HRs) for OS comparing systemic therapy plus local consolidative therapy (LCT) versus systemic therapy alone. **(B)** Cumulative meta-analysis of OS, performed in chronological order of publication, demonstrating the temporal stability of the pooled treatment effect over time.

Pooled analysis demonstrated that the addition of LCT was associated with a significant improvement in overall survival compared with systemic therapy alone (HR = 0.45, 95% CI 0.36-0.56, **Figure 2A**), with moderate heterogeneity observed among studies (I² = 47.4%).

Cumulative meta-analysis revealed a progressively stabilized survival benefit over time, with the pooled effect size converging to an HR of 0.45 by 2025, indicating a consistent and durable association between LCT and improved OS as evidence accumulated (**Figure 2B**).

### 3.3 Progression-free survival

**Figure 3:**
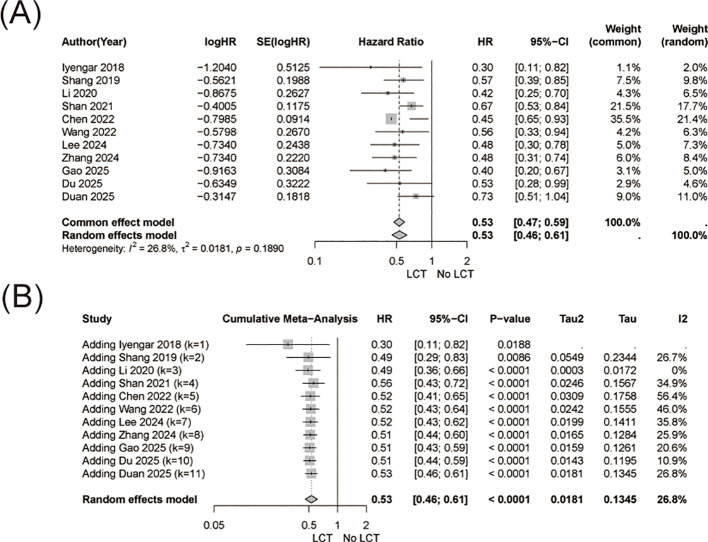
Progression-free survival (PFS) analysis. **(A)** Forest plot showing pooled hazard ratios (HRs) for PFS comparing systemic therapy plus LCT versus systemic therapy alone. **(B)** Cumulative meta-analysis of PFS, illustrating the evolution and stabilization of the treatment effect as additional studies were incorporated.

Similarly, LCT was associated with a significant prolongation of progression-free survival (HR = 0.53, 95% CI 0.46-0.61, **Figure 3A**). Between-study heterogeneity was low to moderate (I² = 26.8%).

Cumulative meta-analysis showed that the PFS benefit emerged early and remained stable over successive studies, with the final pooled HR reaching 0.53 in 2025 (**Figure 3B**).

### 3.4 Objective response rate

**Figure 4:**
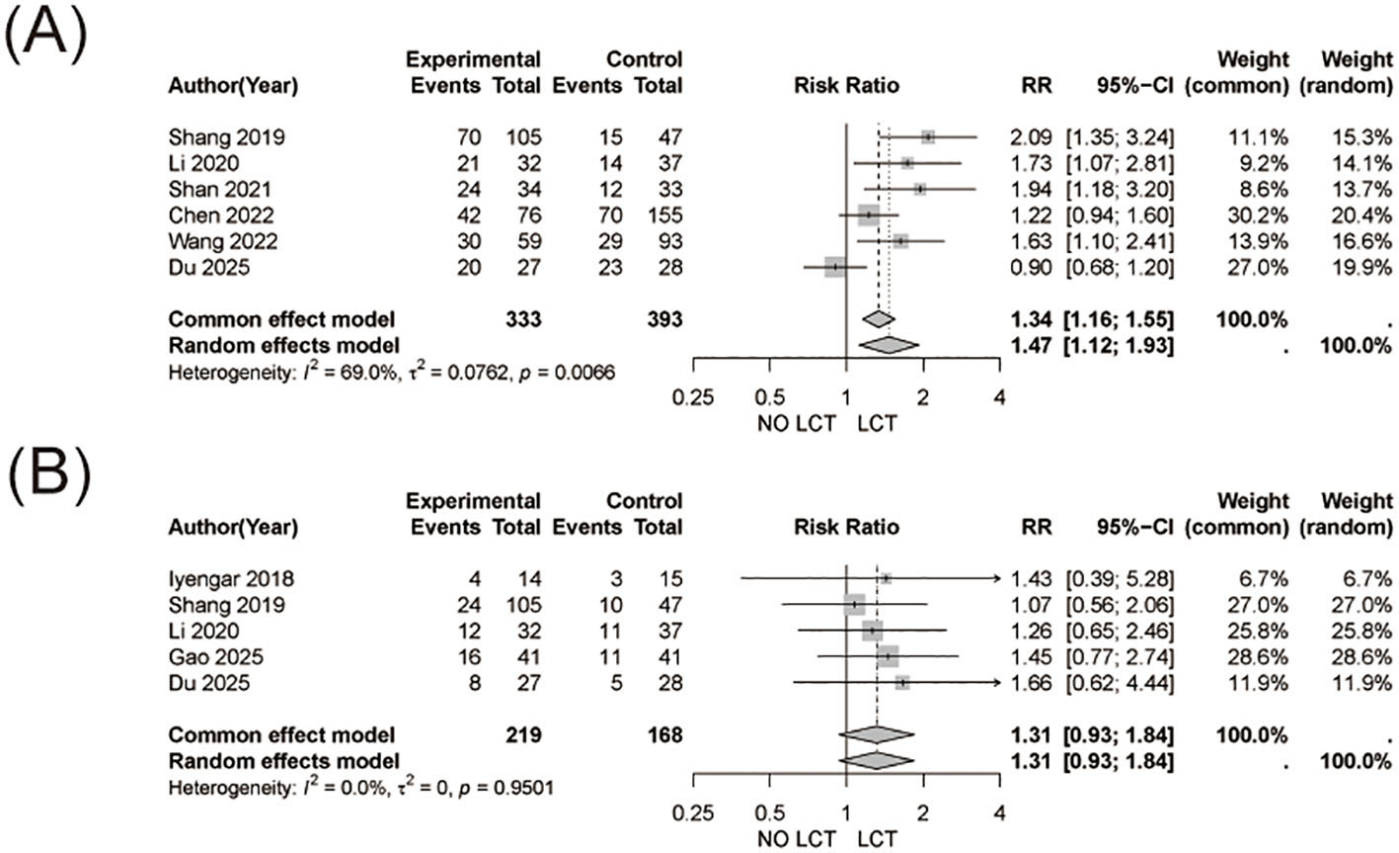
Objective response rate (ORR) and treatment-related adverse events (TRAE). **(A)** Forest plot showing pooled risk ratios (RRs) for ORR comparing systemic therapy plus LCT versus systemic therapy alone. **(B)** Forest plot showing pooled risk ratios (RRs) for TRAE between the two treatment strategies.

The pooled analysis demonstrated a significantly higher objective response rate in patients receiving LCT in addition to systemic therapy compared with systemic therapy alone (RR = 1.47, 95% CI 1.12-1.93, **Figure 4A**). Substantial heterogeneity was observed (I² = 69.0%).

### 3.5 Treatment-related adverse events

No significant difference in the incidence of treatment-related adverse events was observed between patients receiving LCT and those receiving systemic therapy alone (RR = 1.31, 95% CI 0.93-1.84, **Figure 4B**). No heterogeneity was detected across studies (I² = 0%).

### 3.6 Subgroup analyses

Prespecified subgroup analyses demonstrated consistent survival benefits across clinically relevant subgroups.

**Figure 5:**
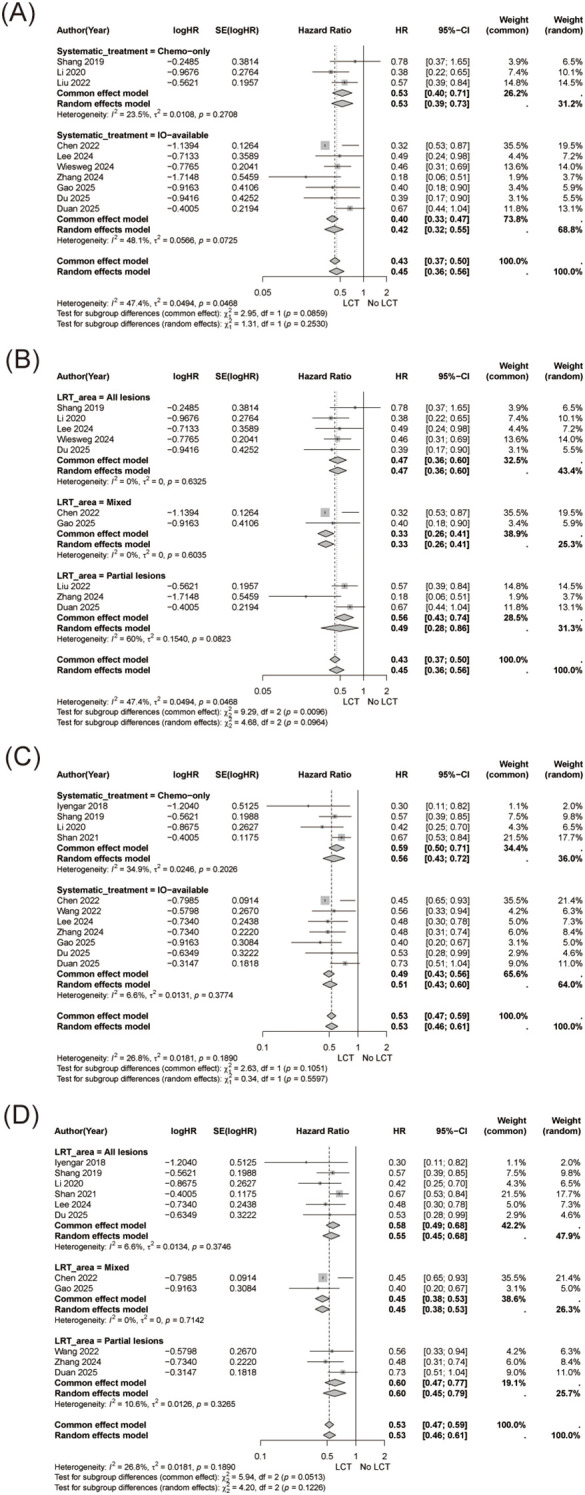
Subgroup analyses for overall survival (OS) and progression-free survival (PFS). **(A)** Subgroup analysis of OS stratified by systemic therapy regimen (immunotherapy-based vs non-immunotherapy regimens). **(B)** Subgroup analysis of OS according to the extent of local treatment coverage (all lesions treated, mixed strategies, and partial lesion treatment). **(C)** Subgroup analysis of PFS stratified by systemic therapy regimen. **(D)** Subgroup analysis of PFS according to the extent of local treatment coverage.

For OS, the benefit of LCT was observed regardless of immunotherapy use, with a numerically greater effect in patients receiving immunotherapy-based regimens (**Figure 5A**). Survival benefits were also observed across different extents of local treatment, including treatment of all lesions, mixed lesions, and partial lesions (**Figure 5B**). Meanwhile, similar patterns were observed for PFS (**Figures 5C, D**).

**Figure 6:**
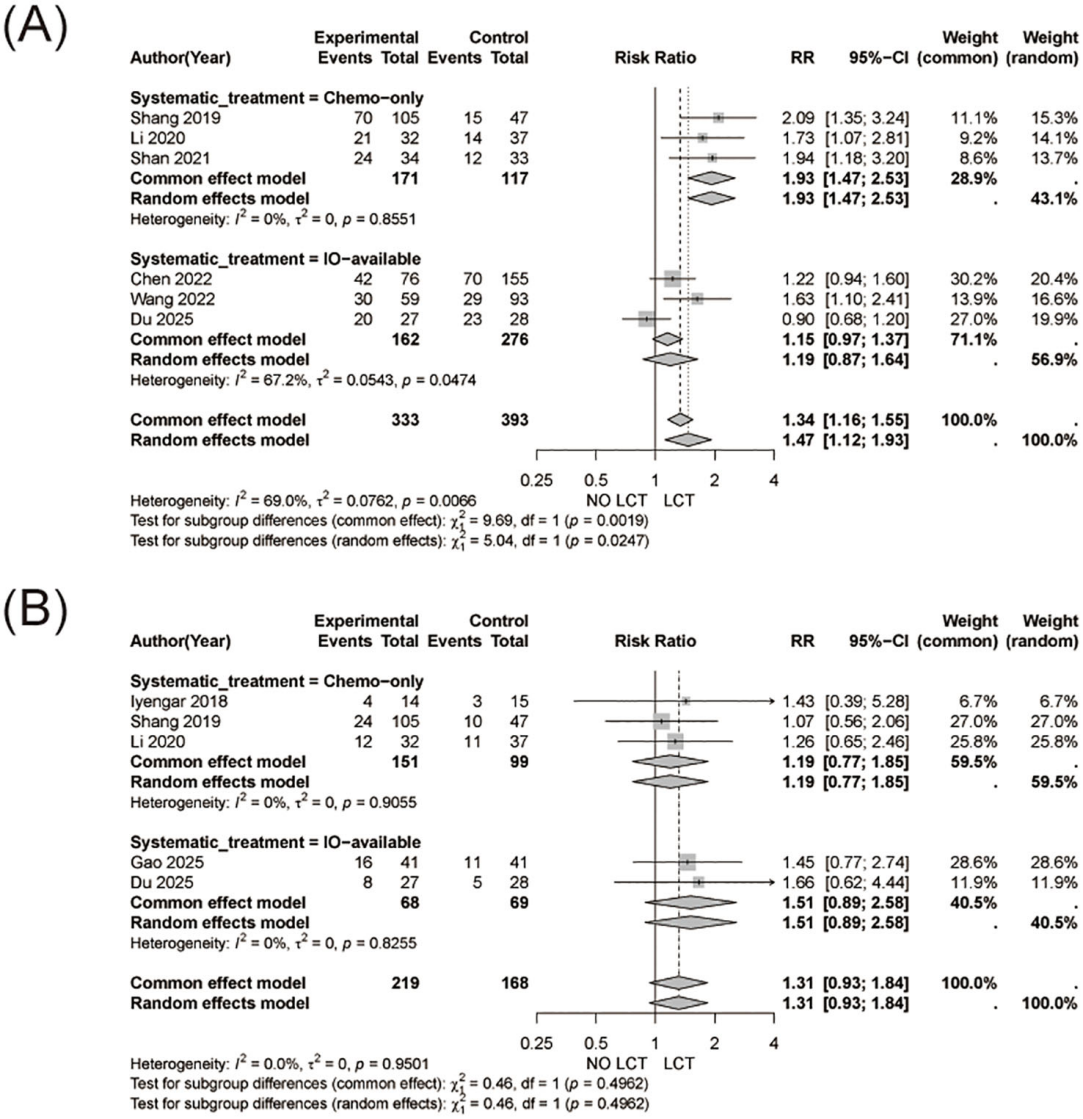
Subgroup analyses for objective response rate (ORR) and treatment-related adverse events (TRAE). **(A)** Subgroup analysis of ORR stratified by systemic therapy regimen. **(B)** Subgroup analysis of TRAE stratified by systemic therapy regimen.

For ORR, a significant benefit of LCT was evident in patients not receiving immunotherapy, whereas no statistically significant difference was observed in immunotherapy-treated patients (**Figure 6A**). And as for different systematic therapy regimens, no significant subgroup differences were observed for TRAE (**Figure 6B**).

### 3.7 Sensitivity analyses for the outcomes

Leave-one-out sensitivity analyses confirmed the robustness of the pooled estimates (**Supplementary Figure 3**). The direction and magnitude of the effect remained consistent across all outcomes, with pooled HRs or RRs varying within narrow ranges (OS: 0.42–0.50; PFS: 0.50–0.54; ORR: 1.37–1.62; TRAE: 1.26–1.41).

### 3.8 Publication bias of the studies

Visual inspection of funnel plots suggested mild asymmetry for OS and PFS (**Supplementary Figure 4**). However, Egger’s regression tests did not indicate significant publication bias for OS (p = 0.58) or PFS (p = 0.66).

## 4 Discussion

Despite the substantial efficacy of targeted therapies in molecularly selected populations and the endorsement of LCT in current clinical guidelines, the supporting evidence has largely been derived from heterogeneous cohorts in which driver mutation status was not consistently distinguished (8). As a result, the clinical value of LCT in patients without actionable oncogenic drivers, who predominantly rely on chemotherapy and or immunotherapy for systemic disease control, has remained insufficiently defined. The present meta-analysis helps clarify the clinical impact of LCT in this underrepresented population of patients with driver-negative oligometastatic non-small cell lung cancer. Across pooled analyses and cumulative meta-analyses, the addition of LCT to systemic therapy was consistently associated with improved survival and disease control, without a corresponding increase in treatment-related toxicity. Importantly, these associations remained stable as evidence accumulated over time, supporting the robustness of the observed findings. Collectively, our results extend previous evidence by demonstrating that even in the absence of actionable driver mutations and in treatment settings not dominated by targeted therapy, LCT retains meaningful clinical value.

Furthermore, the survival benefits associated with LCT persisted in studies in which systemic therapy included immunotherapy, with consistent improvements observed in both overall survival and progression-free survival. These findings help contextualize and refine recent controversies in the field. While the NRG-LU002 trial questioned the routine use of local therapy in the immunotherapy era, the present analysis suggests that the lack of benefit observed in a single randomized setting does not fully negate the potential role of LCT across broader clinical contexts. When evaluated across diverse local treatment modalities and real-world patient populations, LCT remained associated with favorable outcomes. This observation underscores the importance of appropriate patient selection and treatment strategy rather than a uniform acceptance or rejection of local therapy in oligometastatic NSCLC.

Several biological and clinical mechanisms may underlie the observed benefits of LCT in driver-negative oligometastatic NSCLC. From a disease biology perspective, oligometastatic tumors are widely regarded as an intermediate state between localized and widely metastatic disease, in which eradication of all or most macroscopic lesions may delay systemic dissemination and prolong survival (33–35). Local consolidative therapy may reduce overall tumor burden, limit the emergence of resistant subclones, and interrupt ongoing metastatic seeding, thereby enhancing the effectiveness of systemic treatment (36).

In the immunotherapy era, additional mechanisms may contribute to the observed effects. Local treatments such as radiotherapy and ablative procedures can induce immunogenic cell death, promote tumor antigen release, and favorably modulate the tumor microenvironment, potentially amplifying systemic antitumor immune responses (28, 37, 38). This interaction may partly explain why survival benefits were maintained among patients receiving immunotherapy-based regimens. Moreover, subgroup analyses from the present study suggest that the extent of local treatment coverage may influence clinical outcomes, with treatment of all known lesions generally associated with more favorable results than partial coverage. This finding supports the concept that therapeutic intent and completeness of local intervention are critical determinants of clinical benefit.

Differences in study design and treatment strategies may further explain the discrepancies observed between randomized trials and observational evidence. Trials such as NRG-LU002 primarily evaluated radiotherapy-based approaches and applied stringent eligibility criteria and fixed treatment timing, whereas real-world practice often incorporates heterogeneous LCT modalities, flexible sequencing, and individualized multidisciplinary decision-making (11). These distinctions may contribute to differential clinical outcomes and underscore the challenges of directly extrapolating results from highly controlled trial settings to routine clinical care.

Several limitations of this meta-analysis should be acknowledged. First, most included studies were retrospective in nature, which introduces the possibility of selection bias and residual confounding despite methodological adjustments. Second, heterogeneity existed across studies with respect to definitions of oligometastatic disease, LCT modalities, treatment timing, and systemic therapy regimens, all of which may have influenced the pooled estimates. In particular, cumulative meta-analysis was performed across studies with heterogeneous LCT modalities, which may limit the interpretation of modality-specific effects despite providing insight into the temporal stability of the overall treatment effect. Third, the clinical benefit of LCT is unlikely to be homogeneous across all patients with oligometastatic NSCLC. Emerging evidence suggests that both disease burden and tumor biology (e.g., PD-L1 expression) may influence treatment outcomes. In particular, PD-L1 expression has been associated with differential responses to immunotherapy and may modify the incremental value of local treatment. However, due to the lack of patient-level data and inconsistent reporting across studies, these factors could not be formally evaluated in the present analysis. Future prospective studies incorporating biomarker-driven and site-specific stratification are warranted to optimize patient selection for LCT. Last but not least, unlike trials enrolling *de novo* stage IV disease, some included studies focused on postoperative oligometastatic recurrence following initial curative-intent surgery or oligo-residual disease after systemic therapy, which may represent a distinct clinical entity with different tumor biology and prognosis. Nevertheless, the consistent direction of effect across sensitivity analyses and cumulative meta-analyses supports the robustness of the main findings.

Future research should prioritize well-designed randomized trials specifically focused on patients with driver-negative oligometastatic NSCLC, with careful stratification according to systemic therapy backbone, disease extent, and completeness of local treatment. In parallel, translational studies aimed at identifying biomarkers predictive of benefit from LCT may further refine patient selection. As systemic treatment strategies continue to evolve, defining the optimal integration of LCT within multidisciplinary care pathways remains a critical priority to maximize clinical benefit while avoiding unnecessary interventions (39–41).

## 5 Conclusion

In conclusion, this meta-analysis demonstrates that the addition of local consolidative therapy to systemic treatment is associated with improved survival and disease control in patients with driver-negative oligometastatic non-small cell lung cancer, without a significant increase in treatment-related toxicity. By integrating diverse local treatment modalities and focusing on a population in whom targeted therapy does not dominate therapeutic decision-making, our findings provide clinically relevant evidence that helps clarify ongoing controversies regarding the role of local therapy in advanced disease. These results support the consideration of LCT as part of a multidisciplinary treatment strategy for carefully selected patients. Future prospective, biomarker-informed randomized trials are warranted to further refine patient selection, optimize treatment sequencing, and better define the role of local therapy in the immunotherapy era.

**Table 1:** Basic characteristics of the included studies.

Study	Year	Country	Design	Sample size (LCT/No LCT)	Stage distribution (LCT/No LCT)	Disease setting	Oligometastatic definition	Systemic therapy	LCT modality	LCT coverage	Histology (LCT/No LCT)	Staging system version
Iyengar et al.	2018 (6)	US	Randomized, 2-arm, phase 2 trial	14/15	IV for all	Oligo-residual	6 or fewer extracranial lesions (including primary tumor); 3 or fewer metastases within lung and liver	Chemo	Radiotherapy	All lesions	Squamous: 7.1%/20.0% Nonsquamous: 92.9%/80.0%	NR
Shang et al.	2019 (31)	China	Retrospective Study	105/47	I or II*: 57.2%/48.9% IIIA*: 37.1%/36.2% Unknown*: 5.7%/8.5%	Metachronous oligometastatic	5 or fewer metastases	Chemo	Radiotherapy/Surgery/ Ablation/Brachytherapy	All lesions	Squamous: 37.1%/48.9% Nonsquamous: 62.9%/51.1%	AJCC 7^th^
Li et al.	2020 (30)	China	Retrospective Study	32/37	IV for all	Synchronous oligometastatic	5 or fewer metastases. without intracranial extracranial or bone metastases involving spinal cord	Chemo	Brachytherapy	All lesions	Squamous: 58.8%/59.5% Nonsquamous: 41.2%/40.5%	AJCC (IASLC) 8^th^
Shan et al.	2021 (29)	China	Retrospective Study	34/33	IV for all	Synchronous oligometastatic	Primary tumor with a single liver metastasis	Chemo	Ablation	All lesions	Squamous: 62.5%/54.5% Nonsquamous: 37.5%/45.5%	NR
Chen et al.	2022 (28)	China	Retrospective Study	76/155	IV for all	Synchronous oligometastatic	5 or fewer metastases invovling 3 or fewer organs	IO or IO+Chemo	Radiotherapy/Surgery	Mixed	Squamous: 40.8%/38.7% Nonsquamous: 59.2%/61.3%	AJCC 8^th^
Wang et al.	2022 (26)	China	Retrospective Study	59/93	NR	Synchronous oligometastatic: 54.6% Metachronous oligometastatic: 45.4%	3 or fewer metastases	IO or IO+Chemo	Radiotherapy	Partial lesions	Squamous: 28.8%/40.9% Nonsquamous: 71.2%/59.1%	NR
Liu et al.	2022 (27)	China	Retrospective Study	55/67	IV for all	Synchronous oligometastatic	Only mention extracranial metastasis	Chemo	Radiotherapy	Partial lesions	NR	NR
Lee et al.	2024 (25)	Korea	Retrospective Study	74/74	NR	Synchronous oligometastatic	5 or fewer metastases invovling 3 or fewer organs	IO or IO+Chemo	Radiotherapy/Surgery	All lesions	Squamous: 28.4%/32.4% Nonsquamous: 71.6%/67.6%	NR
Wiesweg et al.	2025 (21)	Germany	Retrospective Study	153/65	IV for all	Synchronous oligometastatic	5 or fewer metastases invovling 3 or fewer organs	IO or IO+Chemo or Chemo	Radiotherapy/Surgery	All lesions	NR	AJCC (UICC/IASLC) 8^th^
Zhang et al.	2024 (24)	China	Retrospective Study	43/90	IV for all	Oligo-residual	5 or fewer metastases invovling 3 or fewer organs	IO or IO + other systematic therapy	Radiotherapy/Surgery /Ablation	Mixed	Squamous: 37.2%/45.6% Nonsquamous: 62.8%/54.4%	AJCC 8^th^
Gao et al.	2025 (22)	China	Prospective Study	41/41	NR	Synchronous oligometastatic	5 or fewer metastases invovling 3 or fewer organs	IO or IO+Chemo	Radiotherapy/Surgery	Mixed	Squamous: 41.5%/31.7% Nonsquamous: 58.5%/68.3%	NR
Du et al.	2025 (23)	China	Retrospective Study	27/28	IV for all	Synchronous oligometastatic	5 or fewer metastases invovling 3 or fewer organs	IO+Chemo	Radiotherapy	All lesions	Squamous: 48.1%/46.4% Nonsquamous: 51.9%/53.6%	AJCC 8^th^
Duan et al.	2025 (32)	China	Retrospective Study	84/156	NR	Synchronous oligometastatic	5 or fewer metastases invovling 3 or fewer organs	IO or IO+Chemo	Radiotherapy	Mixed	Squamous: 46.4%/41.0% Nonsquamous: 53.6%/59.0%	NR

The table presents the basic information of 13 included studies. NR, not reported; LCT, local consolidative therapy; AJCC, The American Joint Commission on Cancer; IASLC, The International Association for the Study of Lung Cancer; UICC, The Union for International Cancer Control; IO, immunotherapy; Chemo, chemotherapy; ‘Mixed’ indicates LCT delivered to both the primary tumor and one or more metastatic lesions (not necessarily all metastatic sites); *Indicates postoperative oligometastatic recurrence following initial early-stage disease.

## Data Availability

The original contributions presented in the study are included in the article/**Supplementary Material**. Further inquiries can be directed to the corresponding author.
