# Supplementary material for: Efficacy and safety of local consolidative therapy combined with systemic therapy in driver-negative oligometastatic non-small cell lung cancer: a systematic review and meta-analysis
Source: Front Oncol. 2026 Apr 20;16:1820515. doi: 10.3389/fonc.2026.1820515 (PMC13135962; doi:10.3389/fonc.2026.1820515)
Supplement: Supplementary file 1 [file DataSheet1.docx]

*Supplementary Material*

**Supplementary Table S1. Newcastle-Ottawa Scale (NOS) score of the cohort studies included**

| Study  (Author+Year) | Selection  (Max 4) | Comparability  (Max 2) | Outcome  (Max 3) | Total | Quality level |
| --- | --- | --- | --- | --- | --- |
| Shang2019 | 4 | 1 | 3 | 8 | High |
| Li2020 | 4 | 1 | 3 | 8 | High |
| Shan2021 | 4 | 1 | 2 | 7 | High |
| Chen2022 | 4 | 2 | 3 | 9 | High |
| Wang2022 | 4 | 1 | 3 | 8 | High |
| Liu2022 | 4 | 2 | 3 | 9 | High |
| Lee2024 | 4 | 2 | 3 | 9 | High |
| Wiesweg2024 | 4 | 2 | 3 | 9 | High |
| Zhang2024 | 4 | 2 | 3 | 9 | High |
| Gao2025 | 4 | 2 | 3 | 9 | High |
| Du2025 | 4 | 2 | 2 | 8 | High |
| Duan2025 | 4 | 2 | 2 | 8 | High |

Study quality was evaluated using the Newcastle-Ottawa Scale, which assesses selection (maximum 4 points), comparability (maximum 2 points), and outcome (maximum 3 points). Total scores range from 0 to 9, with scores of 7-9 indicating high quality, 4-6 moderate quality, and ≤3 low quality.


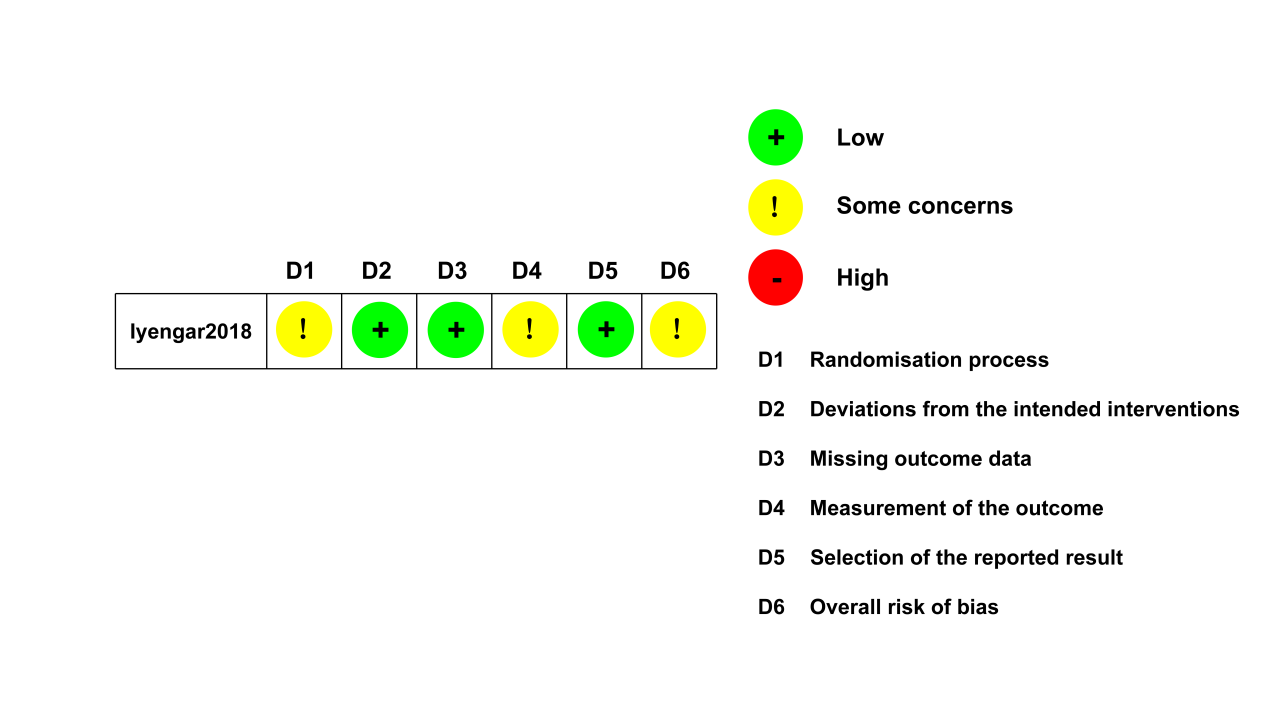


**Supplementary Figure S1. Risk of bias assessment of the included randomized controlled trial using the RoB 2.0 tool**


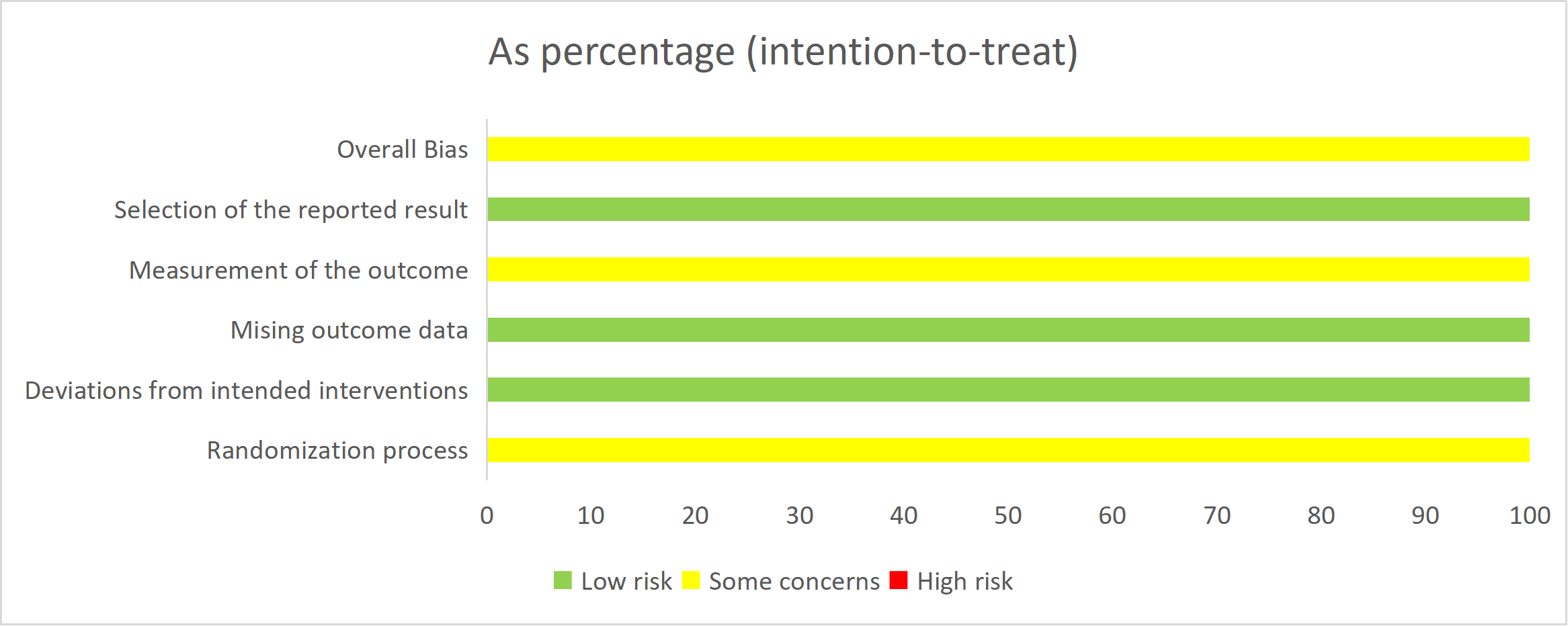


**Supplementary Figure S2.** **Summary of risk of bias domains across included randomized controlled trial**


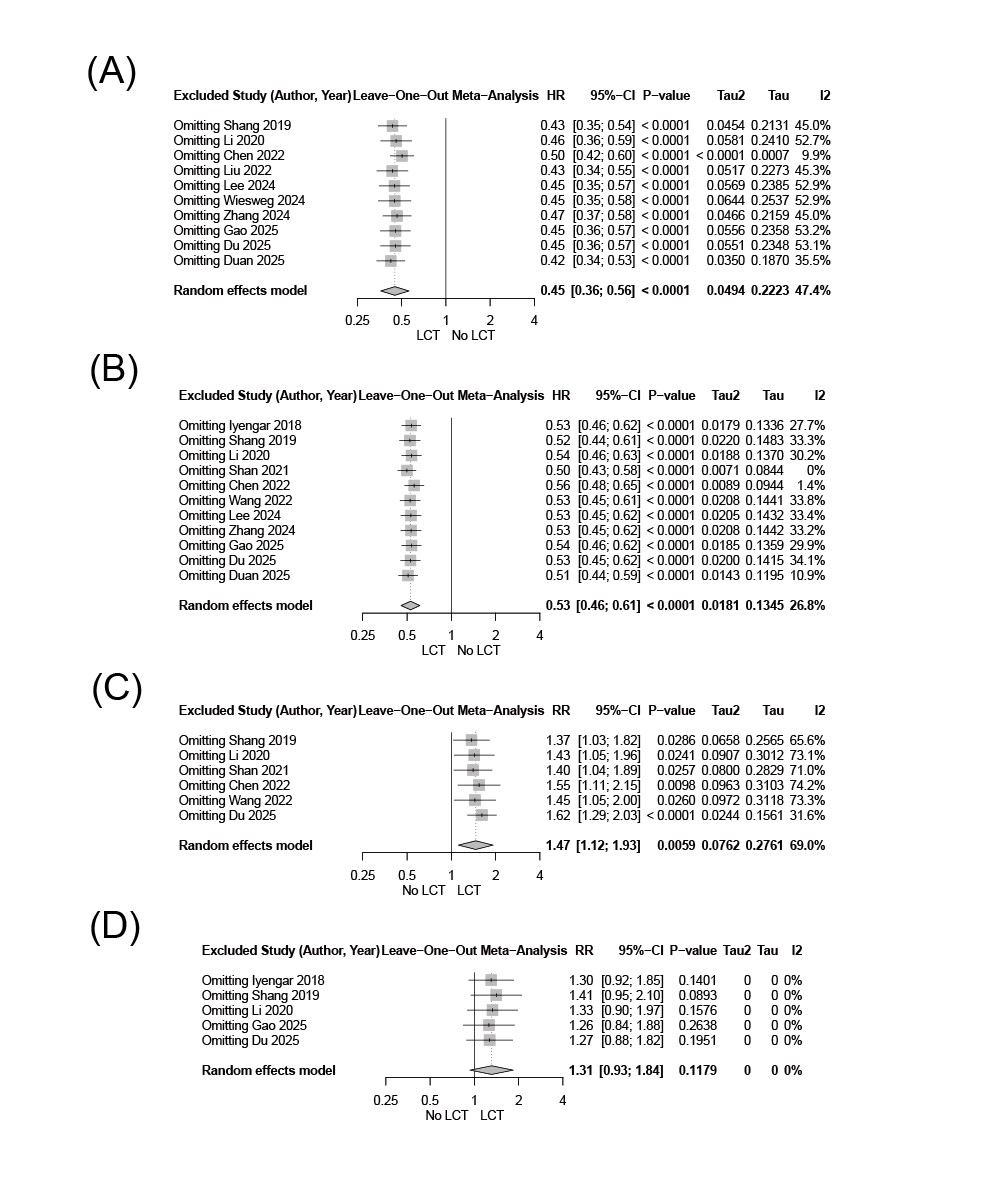


**Supplementary Figure S3.** **Sensitivity analyses**

**
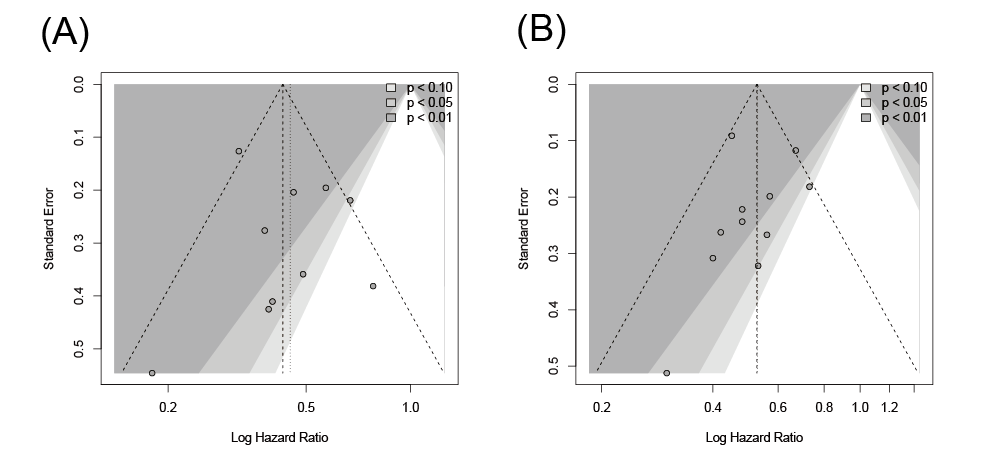
**

**Supplementary Figure S4.** **Funnel plots assessing publication bias**

**Supplementary Figure Legends**

**Supplementary Figure S1. Risk of bias assessment of the included randomized controlled trial using the RoB 2.0 tool**

Risk of bias assessment for individual randomized controlled trials evaluated using the Cochrane Risk of Bias 2.0 (RoB 2.0) tool under the intention-to-treat framework. The study was assessed across six domains: randomization process (D1), deviations from intended interventions (D2), missing outcome data (D3), measurement of the outcome (D4), selection of the reported result (D5), and overall risk of bias. Judgments are presented as low risk (green), some concerns (yellow), or high risk (red).

**Supplementary Figure S2. Summary of risk of bias domains across included randomized controlled trials**

Bar chart summarizing the proportion of studies classified as low risk, some concerns, or high risk for each RoB 2.0 domain under the intention-to-treat framework.

**Supplementary Figure S3. Leave-one-out sensitivity analyses**

Forest plots demonstrating the robustness of pooled estimates for OS, PFS, ORR, and TRAE after sequential exclusion of individual studies.

**Supplementary Figure S4. Funnel plots assessing publication bias**

Funnel plots for OS and PFS, with Egger’s regression tests performed to evaluate small-study effects.
